# Critical congenital heart disease detection in the ANDES: Challenges and opportunities

**DOI:** 10.1016/j.ijcchd.2022.100415

**Published:** 2022-08-17

**Authors:** Kelly Meza, Tania Vasquez-Loarte, J. Franco Rodriguez-Alarcon, Oscar San Roman, Jose Rojas-Camayo, Christian R. Mejia, Monica Medina, Henry A. Zapata, Annamarie Saarinen, Katia Bravo-Jaimes

**Affiliations:** a Universidad Nacional Mayor de San Marcos, Facultad de Medicina de San Fernando, Lima, Peru; b Division of Medicine, Wyckoff Heights Medical Center, New York, USA; c Facultad de Medicina Humana “Manuel Huaman Guerrero”, Universidad Ricardo Palma, Lima, Peru; d Newborn Foundation, Mexico; e Instituto de Investigaciones de La Altura, Universidad Peruana Cayetano Heredia, Lima, Peru; f Translational Medicine Research Center, Universidad Norbert Wiener, Lima, Peru; g Instituto Nacional de Salud Del Niño, San Borja, Lima, Peru; h Division of Neonatology, University of Wisconsin, Madison, Wisconsin, USA; i Newborn Foundation. St Paul, Minnesota, USA; j Ahmanson/UCLA Adult Congenital Heart Disease Center, Los Angeles, California, USA; k Mayo Clinic Florida, Jacksonville, Florida, USA

**Keywords:** Congenital heart disease, High altitude, Andean populations

## Abstract

Critical congenital heart disease (CCHD) represents a challenging problem in global health equity due to the need for specialized surgical or transcatheter intervention within the first year of life. CCHD screening using pulse oximetry (POS) has led to significant improvements in mortality due to early referral and intervention. Andean America represents one of the few regions in the world with increasing CHD deaths and variable POS implementation. In this manuscript, we review the current state of CCHD in Andean America, the challenges and opportunities for developing new POS algorithms that account for high-altitude physiology, data on regional cost-effectiveness supporting POS implementation and outline future directions to achieve equity in CHD care in this region.

## 1 Current state of congenital heart disease in The Andes

**Fig. 1:**
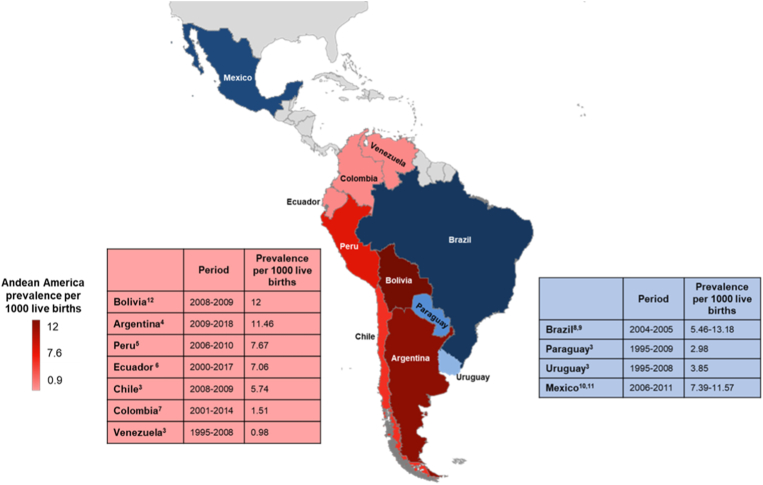
Prevalence of CHD in Andean countries compared with other countries in Latin America. The prevalence in the Andean countries per 1000 live births exhibits significant variation among reports.

It is estimated that 140 million people worldwide live permanently at high altitudes, conventionally defined as at least 2500 m above sea level (masl). Approximately 6 million of them reside in South American countries whose territories include part of The Andes: Venezuela, Colombia, Ecuador, Peru, Bolivia, Chile, and Argentina [1]. In these countries, the prevalence of congenital heart disease (CHD) at birth is probably not different than in the rest of the world (1%), however, there is significant heterogeneity among published studies (Fig. 1) that could be accounted for disparities in access to care, heterogeneous national birth defects surveillance systems, fetal echocardiography availability and high altitude [[2], [3], [4], [5], [6], [7], [8], [9], [10], [11]]. For example, in Colombia, the prevalence was recorded at 1.2 per 1000 live births between 2008 and 2009, whereas in Bolivia, it was estimated at 12 per 1000 and in Chile, 5.7 per 1000 [3,7,12,13]. Overall, approximately 54 000 children are born with CHD every year in Latin America, and only 17 000 of them undergo any treatment. Consequently, a high CHD mortality in the first year of life is found, averaging 108.1 infant deaths per 100 000 children younger than 1 year in Andean America, a strikingly high rate compared to the ones from upper income countries (i.e. United States with 41.5 per 100 000 children younger than 1 year) [14]. These rates vary country to country, with 146.4 infant deaths per 100 000 live births in Mexico [15] and 250 infant deaths per 100 000 live births in Argentina [4]. These facts are supported by the Global Burden of Disease Study, which demonstrated in 2017 that Andean America and Central and Eastern Sub-Saharan Africa are the only regions in the world with increasing CHD deaths [14].

## 2 Critical CHD detection via pulse oximetry: utility and caveats

Critical congenital heart disease (CCHD), defined as CHD requiring surgery or catheter interventions in the first year of life, represents 25% of CHD [16]. CCHD may be ductal-dependent and/or cyanotic, and typically includes left obstructive defects (hypoplastic left heart syndrome, critical aortic stenosis, coarctation of the aorta, aortic arch atresia or hypoplasia), right obstructive defects (tricuspid atresia, pulmonary atresia), outflow tract defects (tetralogy of Fallot, D-transposition of the great arteries, double-outlet right ventricle, truncus arteriosus, Ebstein anomaly and total anomalous pulmonary venous return [16]. Lack of recognition of CCHD in the newborn nursery was estimated to contribute to 50% of deaths among these children prior to the implementation of neonatal pulse oximetry screening (POS) in the United States [17]. In a meta-analysis, POS demonstrated moderate sensitivity (76.3%) and high specificity (99.9%) detecting CCHD, defined as CHD requiring intervention in the first 28 days of life [18]. In 2012, the local health authorities, the Department of Health and Human Services in the United States, the American Academy of Pediatrics (AAP), the American College of Cardiology (ACC), the American Heart Association (AHA), March of Dimes and Newborn Foundation recommended POS be added to the Routine Uniform Screening Panel (RUSP) [19]. State-wide implementation of mandatory policies for neonatal screening for CCHD was associated with a significant decrease of 33% in infant cardiac deaths between 2007 and 2013, compared with states without this policy The goal of CCHD screening is to reduce the number of deaths due to missed or late diagnoses [20]. Moreover, routine use of POS also identifies babies with other diseases (sepsis, pneumonia, persistent pulmonary hypertension of the newborn, etc), whom, if not identified early could potentially lead to significant morbidity or mortality [21]. Therefore, by July of 2018, all states had adopted neonatal POS [22].

**Fig. 2:**
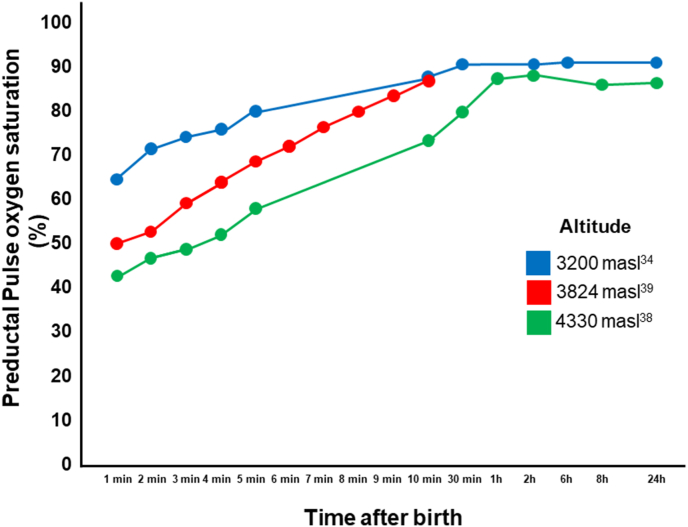
Arterial oxygen saturation in healthy newborns delivered at term in Peruvian cities located above 2500 m above the sea level (masl). Neonatal oxygen saturation increases significantly during the first hour of life during the fetal to the neonatal transition period and stabilizes by the first day of life, even at altitudes as high as 4330 masl.

CCHD screening in the Andes poses significant physiological challenges as, the lower partial pressure of atmospheric oxygen seen with increasing altitudes is more evident at altitudes over 2500 masl [23]. This, associated with genetic variations that provide healthy Andean populations with a blunted hypoxic ventilatory response, higher hemoglobin levels, larger lung volumes, narrower alveolar to arterial oxygen gradients and slightly less hypoxic pulmonary vasoconstrictor response, leads to lower pulse oximetry values across all ages, without affecting oxygen consumption [1,24]. High altitudes have also been associated with a high prevalence of atrial septal defects and patent ductus arteriosus [13,29]. In this context, current POS algorithms, developed and implemented at the sea level, yield a high false positive rate when applied to populations living at moderate or high altitudes [[25], [26], [27], [28]]. Therefore, there is a need to adapt current or develop new algorithms and validate them at these altitudes [29]. This process will need to adequately account for the higher standard deviation [[30], [31], [32], [33], [34], [35]] in oxygen saturation (Table 1), slower remodeling of the pulmonary vasculature [35], and higher pulmonary artery pressures seen with very high altitude [37]. Neonatal oxygen saturation increases substantially during the first hour of life during the fetal to neonatal transition period and stabilizes by the first day of life even at altitudes as high as 4330 masl [34,[38], [39], [40], [41]] (Fig. 2), therefore POS can still be performed starting at the first 24 h of life at high altitudes.

**Table 1:** Pulse-oximetry saturations in healthy neonates at different altitudes.

Author	Altitude (masl)	N	Age (hours)	O_2_Sat range (%)	O_2_Sat mean (%)	O_2_Sat SD (%)	Pre-ductal	Post-ductal	City
Thilo^30^	1610	150	24–48	83–98.2	92.7	0.2	No	Yes	Denver, CO
Bakr^31^	1640	4274	24	91–98	95.4	0.2	Yes	No	Saudi Arabia
Ravert^32^	2073	223	36–48	76–100	93.84	–	Yes	No	Steamboat Springs, CO
Ravert^32^	2073	220	36–48	76–100	94.16	–	No	Yes	Steamboat Springs, CO
Ravert^32^	2392	25	36–48	85–98	94.28	–	Yes	No	Aspen, CO
Ravert^32^	2392	26	36–48	84–99	94.12	–	No	Yes	Aspen, CO
Ravert^32^	2484	59	36–48	89–100	94.53	–	Yes	No	Vail, CO
Ravert^32^	2484	59	36–48	88–100	95.12	–	Yes	No	Vail, CO
Gonzales-Andrade^27^	2820	963	24–48	76–100	92.77	3.03	Yes	No	Quito, Ecuador
Gonzales-Andrade^27^	2820	963	24–48	79–100	93.76	2.83	No	Yes	Quito, Ecuador
Niermeyer^33^	3100	15	24–48	85.2–92.4	88.8	1.8	No	Yes	Leadville, Colorado
Guisbert^34^	3200	109	24	86–97	91	–	Yes	No	Huancayo, Peru
Salas^35^	3665	93	24	–	88.2	3.9	Yes	Yes	La Paz, Bolivia
Gonzalez^38^	4340	37	24	–	87.56	7.23	No	Yes	Cerro de Pasco, Peru

Masl: meters above the sea level, O_2_Sat: oxygen saturation, N: sample size, SD: standard deviation.

## 3 Performance of altitude-modified POS protocols

Due to the unacceptable frequency of false-positive findings in newborns at high altitude, some hospitals have made adaptations to their screening protocol. These strategies include using a lower saturation cutoff for the first screen (85% instead of the recommended 90%), placing the newborn in an oxygen hood to replicate sea level atmospheric oxygen tension, and delaying the screening to 30 h to allow more time for neonatal adaptation [23]. When using a modified protocol including the lower cutoff of 85% and an oxygen hood intervention between the first two screens at 1889 masl, a failure rate of 0.3% was noted, with one newborn with CCHD being missed during the screening [24].

In the absence of standardized cut-off points at high altitude, data from over 2,000 births from a Bolivian Maternity Hospital located at 4,100 masl determined a mean preductal oxygen saturation of 90% (range 77–95) and a mean postductal oxygen saturation of 89% (range 76–89) at 24 h of life. This served to develop a POS algorithm to detect CCHD at high altitude using 84% as a cutoff. Performance evaluation of this cutoff is under development [42]. The use of percentiles has been proposed as an alternative to better classify patients in the field of Pediatrics [[43], [44], [45]]. Additionally, reference values for oxygen saturation at different altitudes have been reported [36], however percentiles for newborn across all habitable altitudes are lacking in the literature, limiting POS application. A study in the region of Yunman, China, with altitudes varying from 267 to 2,202 masl, performed pulse oximetry in 41 097 consecutive neonates and determined that every 1,000-m increase in altitude there was an associated 1.54% decrease in mean oxygen saturation. Whether this holds true at high altitude (>2500 masl) remains to be determined [46] and will be assessed in the ANDES-CHD (*A New algorithm DEtectS critical Congenital Heart Disease*) study.

## 4 Cost-effectiveness of POS in Andean America

The cost-effectiveness of CCHD screening using pulse oximetry compared to clinical examination or cardiac ultrasound alone has been widely demonstrated in North America and the United Kingdom [27,28,47], where it has been implemented for more than a decade. In Latin America, only a few studies examining cost-effectiveness of POS exist.

In Colombia, the costs of POS plus physical examination were US$102; US$7 higher than physical examination alone. The incremental cost-effectiveness ratio (ICER), a metric used in health economics that represents the incremental cost to obtain a certain incremental health effect (in this case an additional identified case of CCHD), was US$100 [48]. Estimated indirect costs per patient (US$775 - US$3802) and out-of-pocket expenses (US$1083 - US$5732), were calculated but significant variation resulted from the need and timing of surgery [49].

In Mexico, the Mexican Committee for the Neonatal Cardiac Screening presented an internal study to the Federal Health Ministry in which they pointed that diagnosing a single child would save the public treasury approximately US$75,000. Furthermore, in the event of detecting and promptly referring 139 newborns in the first 5 years, the total cost of investment of a Federal Program would be covered [50]. Additionally, a cost-effectiveness analysis comparing physical examination with physical examination plus POS using a decision tree model estimated that POS detects 32 additional CCHD cases early for every 10 000 newborns screened. The incremental cost of applying POS to each newborn was US$8.22. Using an expected incremental cost for each additional case detected early was US$3143.29, the conclusion was that POS in addition to physical examination was cost-effective in the Mexican setting [51].

Cost-effectiveness studies in other Andean American countries are needed, however we recommend considering the following issues. First, reporting effectiveness in terms of quality-adjusted life years (QALYs), disability-adjusted life years (DALYs) or health-adjusted life years (HALYs) which is a population health measure that permits morbidity and mortality to be simultaneously described within a single number, this number could be used at the macro and micro levels; the former to track population health and monitor population-based interventions and the latter to assess the effectiveness of preventive and therapeutic interventions [[52], [53], [54]].

Second, identifying accessible and reliable sources of information, possibly involving official data from each country's Ministries of Health. Third, developing surveys assessing indirect costs and the “impact on quality of life” secondary to a false positive result. Even though prior studies demonstrate that POS false-positive results might not elicit parental psychosocial risk of harm [16], we cannot extrapolate this to all the various rural, remote and under-resourced settings in the Andean American context, since many parents might have to wait for their newborns to be referred to a more complex hospital in order to access echocardiography or other specialized testing.

## 5 Considerations for POS implementation in Andean America

The United States' successful experience with POS implementation provides an example of the degree of active involvement of stakeholders needed to achieve this goal, including social, organizational, economic, advocacy and public policy components. In Andean America it is of utmost importance to consider multi-pronged interventions that address (1) parental empowerment, using culturally-competent, longitudinal family education regarding the natural history and complications of CCHD and the importance of neonatal POS, (2) training of medical and nursing staff as well as community health care workers who may be attending deliveries in remote areas, (3) optimization of referrals systems to specialized congenital heart centers with a clearly defined coordination structure, (4) development of information systems to better allocate resources, coordinate with programs and services, and follow-up patients, (5) transparent resource management by health authorities, and (6) leveraging remote technology and artificial intelligence to bridge the gap in echocardiography availability for both testing and follow-up care, developing automatic deep-learning imaging algorithms to simplify and improve the efficiency of ultrasound-CHD detection [55,56].

**Fig. 3:**
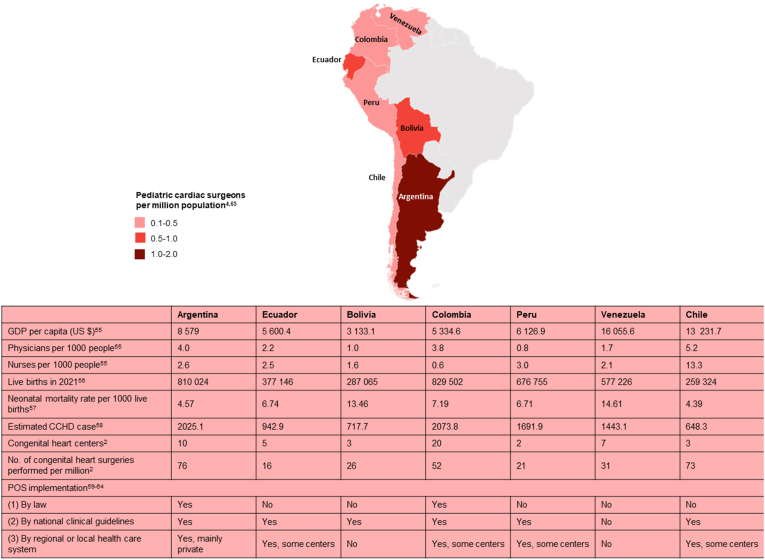
Factors influencing CCHD detection and CHD care across Andean America region. Significant discrepancies exist in the amount of pediatric cardiac surgeons, CHD centers, congenital heart procedures, and POS implementation efforts.

The above interventions should be accompanied by holistic multisectoral efforts towards equitable CHD care in this region since significant disparities exist. The number of congenital heart centers varies among the Andean countries, with Colombia and Argentina having the most (around 7 to 10) and Peru, Chile, and Bolivia having the fewest (Peru, Chile, and Bolivia) (around 2 to 3). Furthermore, according to the Cardiothoracic Surgery Network database, Argentina had 1-2 pediatric cardiac surgeons per million people, compared to 0.5–1 pediatric cardiac surgeons per million people in Ecuador and Bolivia, while Colombia, Venezuela, Peru, and Chile had the lowest number of pediatric cardiac surgeons per million people (0.1–0.5) [[57], [58], [59], [60],^72^]. (Fig. 3). On one hand, a significant number of CHD care initiatives are being performed in countries like Colombia, Argentina, and Chile. In the former, a National Congenital Heart Disease Program coordinates referral, transfer, treatment, and follow-up of children with CHD who lack insurance coverage since 2008. This program has achieved an increase of more than 40% in the number of diagnosed patients and a decrease of 84% in the number of patients on the surgical waiting list [61]. Similarly, POS has been recommended by the Ministries of Health of Colombia [62], Argentina [63], Bolivia [42], Ecuador [64] and Chile [65] through their clinical practice guidelines, however implementation has been partially implemented by independent initiatives probably due to lack of accompanying legislation and dedicated, sustainable funding [61,66]. At this point, it is reasonable to anticipate that POS implementation will be required to achieve the 2030 United Nations Sustainable Development Goals (SDGs), where SDG 3.2 aims to reduce neonatal mortality rate to at least 12 per 1000 live births and end preventable deaths of newborns and children under 5 years of age and SDG 3.4 aims to reduce mortality due to non-communicable diseases, where is CHD is included [67]. However, Argentina and Peru achieved a reduction in neonatal mortality rates to 4.5 and 6.7 per 1000 live births, respectively using strategies directed towards improving obstetric and neonatal care without having national POS implementation [61,[68], [69], [70]]. We believe though, that in order to decrease surgical CHD mortality in Andean America, early diagnosis and referral using national POS implementation accompanied by an adequate increase in surgical and nursing CHD capacity are sorely needed [71,72].

## 6 Future directions

As we continue addressing the challenges imposed by the high CHD mortality in Andean America, we need to timely address several factors that will be important for the future development of children with CHD as they age and require different services. First, the development of national surveillance systems supported by the Ministries of Health in collaboration with other ministries will be needed, either focused on CHD or associated with other congenital birth defects which ultimately will strengthen the health system in concordance with the SDGs [73,74]. To date, only Brazil, Argentina and Colombia are the countries in Andean America with national surveillance systems including CHD [74]. Utilizing existing registries such as the Perinatal Information System supported by the Pan-American Health Organization, and those from collaborative groups such as the Latin-American Collaborative Study of Congenital Malformations (ECLAMC) and International Quality Improvement Collaborative (IQIC), can be useful starting points for estimating CHD incidence and planning Cardio-Obstetrics care in women with CHD [3,75,76]. Quality improvement initiatives that elevate consistency in definitions, data collection systems, monitoring, training, technology integration (i.e. geolocalization), education (with culturally appropriate resources), outcomes, and other unintended consequences in these surveillance systems should be considered [76]. Second, the creation of a robust network involving patients, primary care providers (including rural physicians), cardiologists, social workers and other team members involved in the care of children and adults with CHD will serve to efficiently allocate resources for prevention, treatment, education and research. This network could also serve as a basis for Cardio-Obstetrics care and transition of care from pediatric to adult CHD providers [77].

**Fig. 4:**
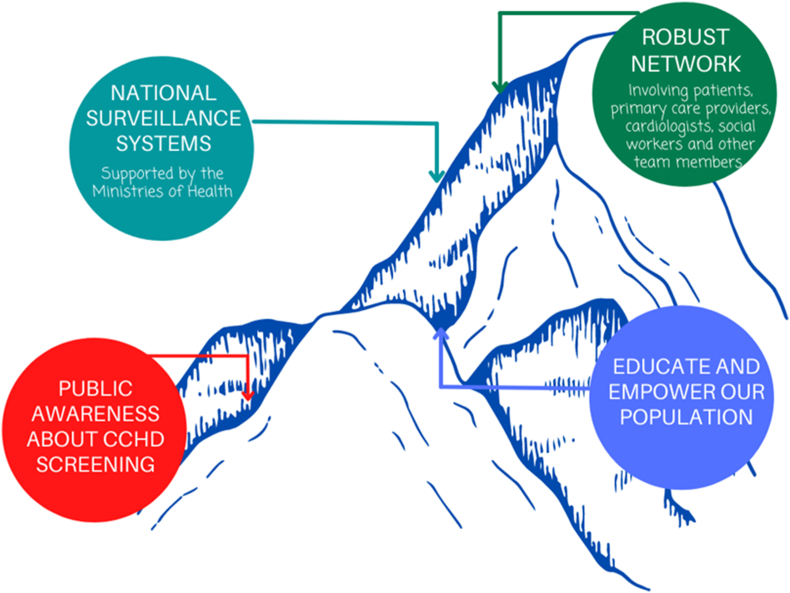
Future POS implementation directions in Andean America. These include the creation of national surveillance systems, robust networks, increased public awareness about CCHD screening, and population education and empowerment.

Finally, raising public awareness about CCHD screening; educating, empowering and engaging patients and emphasizing the need for longitudinal CHD care will need to be implemented as these initiatives are implemented (Fig. 4) [78].

## Disclosures

None.

## Funding

Self-funded.

## Declaration of competing interest

The authors declare that they have no known competing financial interests or personal relationships that could have appeared to influence the work reported in this paper.
